# Epoxidation of Carbon Nanocapsules: Decoration of Single-Walled Carbon Nanotubes Filled with Metal Halides

**DOI:** 10.3390/nano8030137

**Published:** 2018-02-28

**Authors:** Lucia D’Accolti, Agnieszka Gajewska, Magdalena Kierkowicz, Markus Martincic, Angelo Nacci, Stefania Sandoval, Belén Ballesteros, Gerard Tobias, Tatiana Da Ros, Caterina Fusco

**Affiliations:** 1 Department of Chemistry, University of Bari “A. Moro”, Via Orabona, 4, 70126 Bari, Italy; lucia.daccolti@uniba.it (L.D.); angelo.nacci@uniba.it (A.N.); 2 INSTM Unit of Trieste, Department of Chemical and Pharmaceutical Sciences, University of Trieste, Via L. Giorgieri 1, 34127 Trieste, Italy; agnikus@gmail.com; 3 Institut de Ciencia de Materials de Barcelona (ICMAB-CSIC), Bellaterra, 08193 Barcelona, Spain; mkierkowicz@gmail.com (M.K.); markus.martincic@gmail.com (M.M.); ssandoval@icmab.es (S.S.); 4 Catalan Institute of Nanoscience and Nanotechnology (ICN2), CSIC and The Barcelona Institute of Science and Technology, Campus UAB, Bellaterra, 08193 Barcelona, Spain; belen.ballesteros@icn2.cat; 5 CNR-ICCOM, Department of Chemistry, University of Bari, Via Orabona, 4, 70126 Bari, Italy

**Keywords:** SWCNT, TFDO, epoxidation, nanocapsules filling

## Abstract

Methyl(trifluoromethyl)dioxirane (TFDO) can be used for the oxyfunctionalization of SWCNTs filled with NaI and LuCl_3_ under mild conditions. The chosen metal halides are of interest for theranostics, both for imaging and therapy when in their radioactive form. The applied functionalization methodology does not require metal catalyst, preserves the integrity of the nanotubes during treatment, avoiding the release of the filling material. In this way, epoxidation can be considered as an efficient methodology for the functionalization of carbon nanocapsules, where the traditional harsh oxidation conditions by acids are not applicable.

## 1. Introduction

Single-walled carbon nanotubes (SWCNTs) present unique physical, chemical properties and, in recent times, have emerged as a novel class of multifunctional nanomaterials, with application as sensors and biosensors, [1,2,3,4,5] nanoprobes [6] and as potential drug delivery vehicles for therapeutic agents in nanomedicine [3,4]. In fact, their surface area can be exploited to add different biological molecules, so as to create appropriate systems for drug delivery [3,4]. The encapsulation of compounds inside nanotubes offers a new type of sustained nanocomposites for diverse range of application [7,8,9,10,11]. For instance, CNTs filled with paramagnetic metal ions may be managed by an external magnetic field, which would allow the controlled delivery of drugs loaded in the CNT cavities.

One of the most important strategies to render CNTs biocompatible and dispersible in aqueous solutions is their surface modification, through which functional groups are covalently linked to the walls or to ends of the CNTs [3,4].

As tool for functionalization, the oxidation of CNTs introduces functional groups that could be allowed to react with other compounds, providing new structures. For example, in bio-related applications, CNTs are usually treated with strong acids, generating functional groups (predominantly carboxylic acid) on the surface and the tips of CNTs, which allows the dispersion of CNTs in aqueous solution [12]. This approach is really harsh and it is commonly used to open the tips of the tubes.

Mild oxidation methods that can generate oxygen functional groups on CNTs almost maintaining their pristine characteristics is challenging for the preparation of a versatile platform. Oxidation with strong acids can strongly alter the graphitic structure and create defects and holes in the structures [13,14]. In some applications, non-destructive methods are essential to introduce groups without heavily modifying the CNTs surface. This is for instance the case when CNTs are loaded with radiotracers. The creation of structural defects would result in leakage of the radionuclides from the interior of the CNTs and their accumulation into high affinity organs [10].

**Figure 1:**
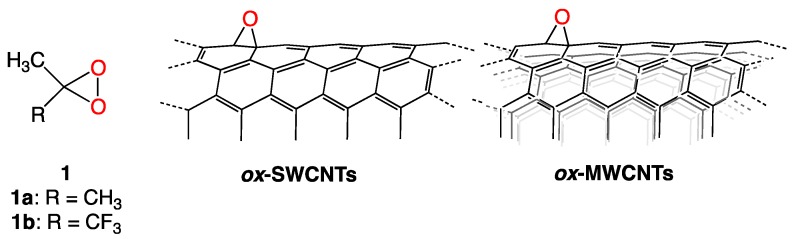
Chemical structure of **1**, *ox*-SWCNTs and *ox*-MWCNTs.

Dioxiranes (**1**, Figure 1) are known to be useful in the selective oxidation of a many organic compounds under mild conditions [15]. Their application for the synthesis of dimethyldioxirane (**1a**; DDO) [16,17] and methyl(trifluoromethyl)dioxirane (**1b**; TFDO) [18,19,20] allowed to prepare important products in organic synthesis [21,22,23,24]. TFDO (**1b**) is an efficient, selective, and environmentally sustainable oxygen-transfer reagent, which can be employed at low temperatures (as at least 0 °C) in the epoxidation of C=C double bonds. Its use is a valuable option to avoid peracids in epoxidation, thanks to improved reaction rates and capability to give epoxidation under neutral conditions [15,21,22,23,24].

We have shown that the oxidation of SWCNTs [25] and MWCNTs [26] into their epoxy group derivative under mild conditions can be obtained using isolated TFDO (**1b**, Figure 1).

Considering these achievements, it appeared convenient to explore the surface modification of SWCNTs filled with non-radioactive metal halides (NaI and LuCl_3_), with the aim of proving that the whole process is properly applicable also to close-ended filled SWCNTs (referred to as “carbon nanocapsules” [11]) without causing leakage of the encapsulated compounds. If so, the developed approach could then be employed with nanocapsules loaded with the respective radionuclides (for instance Na^125^I and ^177^LuCl_3_) of interest for biomedical imaging and therapy, thus increasing the orthogonality of the functionalization process and opening new scenarios in their derivatization.

This study is the natural progression of the previous works [25,26] in which the efficacy of the functionalization on empty single walled and multiwalled nanotubes by means of dioxirane epoxidation has been demonstrated. Herein, we report the surface decoration of biomedically relevant metal halide-filled SWCNTs using the powerful dioxirane **1b**. This method preserves the integrity of the nanotube structure and allows for their successive derivatization (epoxide opening) by means of a diamine [25,26].

The decorated SWCNTs were characterized by Fourier transform infrared (FT-IR), thermogravimetric (TGA), Raman analysis, and transmission electron microscopy (TEM).

## 2. Materials and Methods

The procedure of methyl(trifluoromethyl)dioxirane preparation (**1b**) has been already reported [21]. TGA of pristine and functionalized CNTs were performed on Q500 (TA Instruments) under N_2_ atmosphere by equilibrating at 100 °C, and following a ramp of 10 °C min^−1^ up to 800 °C with a flow rate of 90 mL min^−1^. Less than 1 mg of compound per each analysis was required. Reported plots are an average of at least two measurements. The degree of functionalization as µmol/g was calculated using the following formula:

$$\frac{µ\mathrm{mol}}{g}=\frac{\mathrm{weight}\;\mathrm{loss}\;\left(\%\right)\cdot 10000}{\text{Molecular weight of functional group}}$$

Raman spectra were recorded with a Renishaw inVia Raman microscope (50 objective) using 633 nm He/Ne laser (17 mW) at room temperature with a low laser power.

Infrared analyses were carried out in the 400–4000 cm^−1^ range (4 on 32 scans) with Fourier-transform infrared (FTIR) spectrometers (PerkinElmer Spectrum BX and System2000).

HAADF-STEM and HRTEM images were acquired using an FEI Tecnai G2 F20 microscope operated at 200 kV.

Common commercial solvents and reagents of high purity were employed. Commercial triple salt 2KHSO_5_·KHSO_4_·K_2_SO_4_ (trade name, Caroat^®^), a gift from Peroxid-Chemie (Degussa, Germany) was our source of potassium monoperoxysulfate for the synthesis of TFDO, **1b**. Chemical vapour deposition SWCNTs (Elicarb^®^) were provided by Thomas Swan & Co. Ltd. (Consett, UK) with an average diameter of 2.1 nm (value provided by the supplier). LuCl_3_@SWNCTs were prepared following the protocol previously described [27]. Briefly, pristine SWCNTs were steam treated at 900 °C for 4 h followed by an HCl wash [28], which leads to a median length of 420 nm. The so obtained SWCNTs were filled with LuCl_3_ by molten phase capillary filling, by annealing a mixture of SWCNTs and LuCl_3_ at 950 °C for 12 h under vacuum. The resulting sample consists on closed-ended [29] and filled SWCNTs containing a large excess of non-encapsulated LuCl_3_, which was removed from the sample of using a Soxhlet with dialysis sack system [27]. Similarly, NaI was encapsulated by molten phase capillary filling, by annealing a mixture of steam purified SWCNTs and NaI (1:10 wt. ratio) at 900 °C in a silica ampoule under vacuum. The external NaI was removed by washing the collected sample with water, under reflux during 24 h. The powder was collected by filtration on top of a polycarbonate membrane (0.2 µm pore) and subsequently dried overnight at 60 °C.

*Preparation of ox-NaI@SWCNTs* (**3a**). An aliquot of the standardized cold solution of dioxirane **1b** (0.6 M, 2.5 mL, 1.2 mmol) in 1,1,1-trifluoropropanone (TFP) was added to a suspension of pristine NaI@SWCNTs **2a** (16.34 mg) in cold (0 °C) CH_2_Cl_2_ (20 mL), previously sonicated for 30 min. The reaction was stirred at room temperature for 12 h. The final product was washed many times with CH_2_Cl_2_ followed by MeOH and dried in vacuum to afford 19.7 mg of oxidized product **3a**. FT-IR (spectrum in KBr pellets): ν 3340, 1715, 1552, 1086 cm^−^^1^.

*Preparation of ox-LuCl_3_@SWCNTs* (**3b**). The analogue procedure was used with a suspension of pristine LuCl_3_@SWCNTs **2b** (17.87 mg) in CH_2_Cl_2_ (20 mL), previously sonicated for 30 min, obtaining **3b** (22.3 mg). FT-IR (spectrum in KBr pellets): ν 3340, 1725, 1550, 1083 cm^−^^1^.

*Functionalization of ox-MX@SWCNT* (**3**) *with 1,6-Hexa-Methylendiamine.* Nanotubes *ox*-NaI@SWCNTs **3a** (17.8 mg) were dispersed in 1,6-hexamethylenediamine (3.1 g) and the mixture was heated to reflux at 140 °C for 12 h under N_2_. Then the mixture was diluted with MeOH (20 mL) and filtered by using a 0.2 μm PTFE membrane filter and the solid was washed many times with CH_2_Cl_2_ and MeOH to remove unreacted 1,6-hexamethylenediamine. The resulting material was dried under vacuum to give **4a** (23 mg). FT-IR (spectrum in KBr pellets): ν 3367, 2914, 2845, 1552, 1450, 1381, 1235, 1214, 1090 cm^−1^. The same procedure was applied for the functionalization of **3b** with 1,6-hexamethylenediamine. The degree of functionalization was obtained by TGA, comparing the value of weight loss of **4a** and **4b** with the ones of pristine filled SWCNTs (**2a** and **2b**) and it results 9.7% for *f*-NaI@SWCNT (**4a**) and 11.9% for *f*-LuCl_3_@SWCNT (**4b**). FT-IR (spectrum in KBr pellets): ν 3271, 2917, 2850, 1560, 1449, 1379, 1235, 1214, 1089 cm^−^^1^.

## 3. Results and Discussion

**Figure 2:**
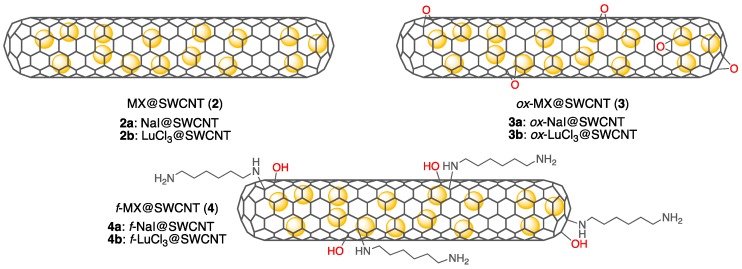
Schematic representation of MX@SWCNTs **2**, *ox*-MX@SWCNTs **3** and *f*-MX@SWCNTs **4**.

The covalent functionalization of SWCNTs filled with NaI (NaI@SWCNTs) or LuCl_3_ (LuCl_3_@SWCNTs) (**2a**–**b**, Figure 2) was performed with dioxirane **1b**. The metal halide nanocapsules, MX@SWCNTs (where MX indicates either NaI or LuCl_3_), were prepared by molten phase filling at high temperature, following the protocols detailed in the experimental section, thus leading to samples of closed–ended filled SWCNTs [29].

### 3.1. Oxidation of MX@SWCNT with Isolated TFDO *(**1b**)*

The as-prepared MX@SWCNTs were treated with **1b** mainly obtaining the introduction of epoxide groups onto the SWCNTs sidewalls. A stock solution (0.5–0.6 M) of **1b** can be prepared by the procedure previously described [21] from 1,1,1-trifluoropropanone (TFP) and stored at −20 °C for months.

**Scheme 1:**
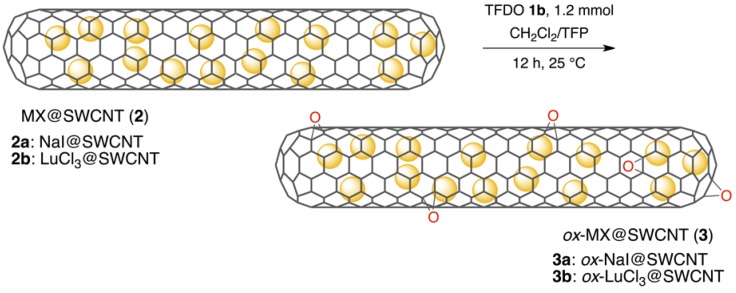
MX@SWCNTs epoxidation with TFDO (**1b**).

In a typical reaction, 16 mg of MX@SWCNTs (**2a** or **2b**) were dispersed in CH_2_Cl_2_ (dry, 20 mL) by sonication for 30 min. To this suspension, dioxirane (**1b**) was added and the mixture was stirred at 25 °C for 12 hours. Scheme 1 shows the decoration of the external surface of **2** by means of **1b** [25].

Subsequently, *ox*-MX@SWCNTs **3** were recovered by filtration on a 0.2 μm PTFE membrane filter and systematically treated several times with CH_2_Cl_2_ and MeOH by filtration/washing workup procedure. Then, the oxidized nanotubes **3** were dried under vacuum.

### 3.2. Post-Functionalization of ox-MX@SWCNTs ***3*** with 1,6-Hexamethylenediamine

Epoxide functional groups provide a large versatility for further subsequent derivatization. Due to their ability to undergo nucleophilic attack with subsequent ring opening, this is a starting point for a wide range of CNTs modifications. As a matter of fact, this process is designed to chemically attack the CNTs sidewall, allowing further covalent derivatization, as already reported and demonstrated by model reaction [25,26,30].

The generation of hydroxyl and amine functional groups on the CNTs allows the preparation of highly functionalized samples.

**Scheme 2:**
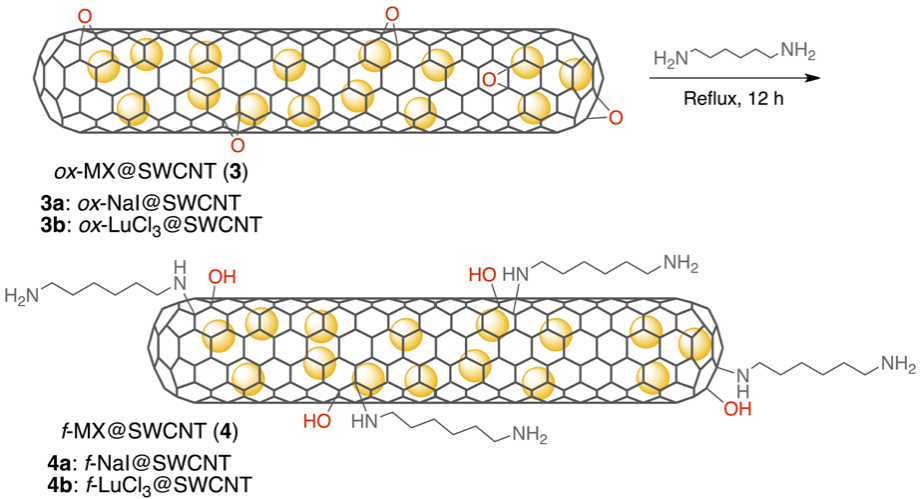
Functionalization of *ox*-MX@SWCNT **3** with 1,6-hexa-methylendiamine.

The reaction between *ox*-MX@SWCNTs **3** and an aliphatic diamine (1,6-hexamethylendiamine) was performed. This reagent was chosen to introduce multimodality to the obtained constructs (Scheme 2).

To obtain this, **3a** is dispersed in excess of 1,6-hexa-methylenediamine to exclude the formation of cross-linking among the tubes and heated at reflux for 12 h under inert atmosphere (N_2_). Afterwards, the resulting suspension is subjected to sonication/washing cycles and then filtered on a 0.2 μm PTFE membrane.

**Figure 3:**
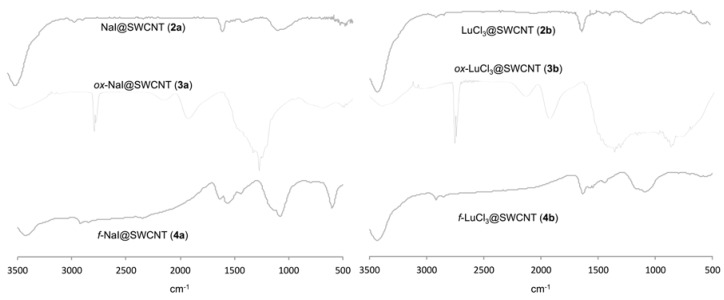
FT-IR spectra of MX@SWCNT **2**, *ox*-MX@SWCNT **3**, (light grey) and *f*-MX@SWCNT **4**.

IR analysis of **4** resulted useful to check the functionalization and the epoxide opening (Figure 3). An NH stretch band is present at 3271 cm^−1^, together with a band at 3367 cm^−1^ related to the NH_2_ symmetric stretch. Moreover, signals at 1450 and 1381 cm^−1^, attributable to the bending band of the CH_2_ group, were detected, and signals at 1235 and 1214 cm^−1^, attributed to C–N bending, were also found [25].

**Figure 4:**
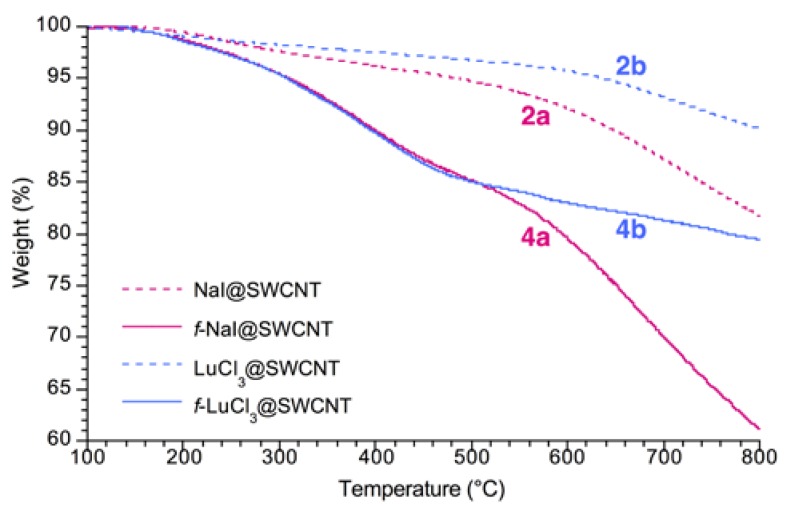
TGA curves of **2** (MX@SWCNTs) and **4** (*f*-MX@SWCNTs) under flowing nitrogen.

The presence of covalently linked organic moieties on *f*-MX@SWCNTs walls was investigated by thermogravimetric analysis, under nitrogen atmosphere in the temperature range from 100 to 800 °C. The TGA curves of MX@SWCNTs and *f*-MX@SWCNTs, i.e., before the chemical treatment and after epoxidation/amination, are depicted in Figure 4. Samples after modification (*f*-MX@SWCNTs) showed a weight loss starting at temperatures above 150 °C with respect to not modified MX@SWCNTs. At 500 °C the mass loss, attributable to the degradation of covalently attached organic moieties, was of 11.9% for the *f*-LuCl_3_@SWCNTs and 9.7% for *f*-NaI@SWCNTs corresponding to a calculated degree of functionalization (f) of 900 µmol/g and 730 µmol/g, respectively.

**Figure 5:**
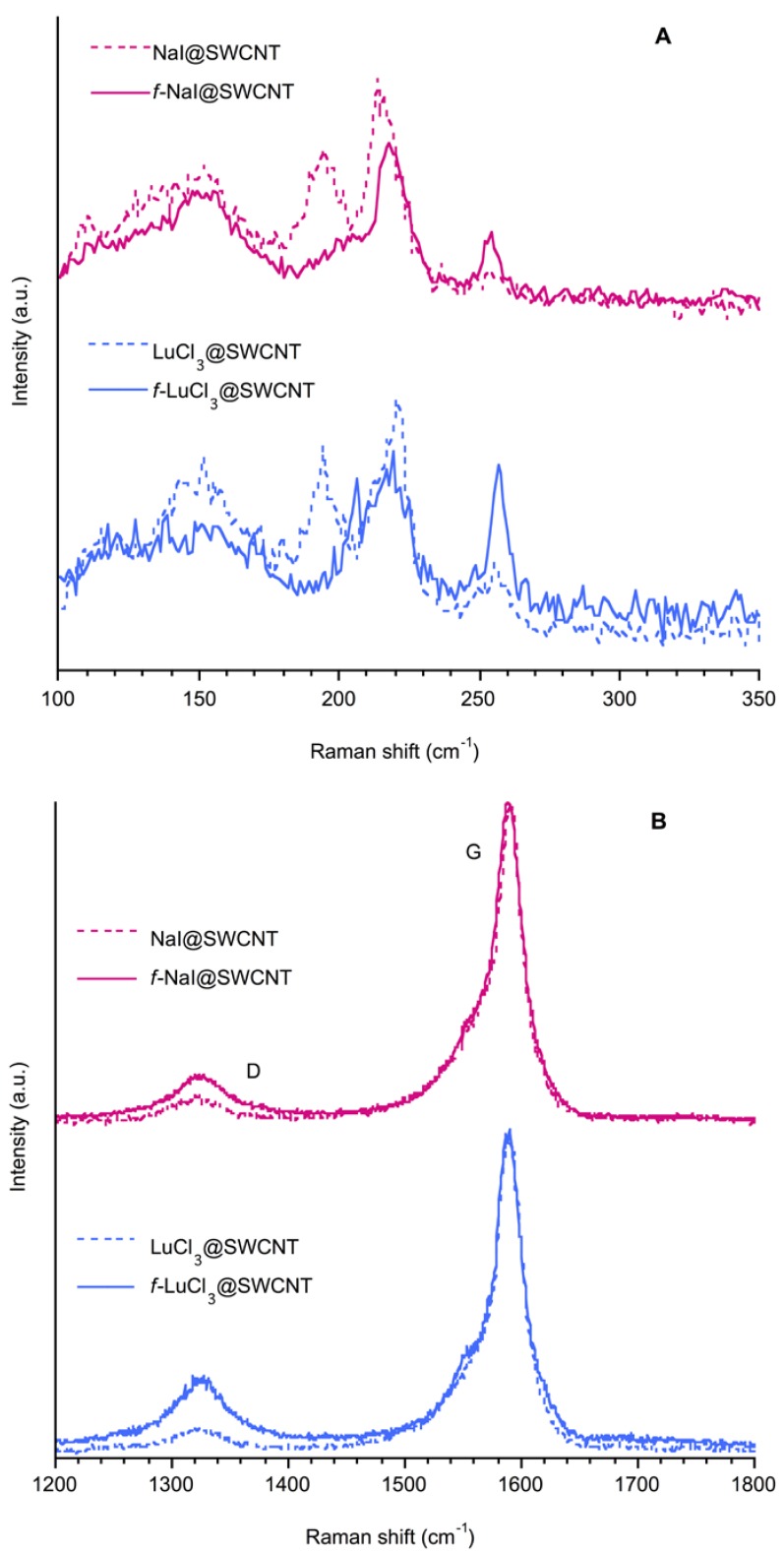
Raman spectra of **2** (dashed lines) and **4** (solid lines) with a 633 nm laser. Panel **A**: RBMs mode; Panel **B**: D mode and G mode. Spectra are normalized for ease of comparison.

Raman spectroscopy represents an effective technique to characterize SWCNTs. The typical Raman spectrum shows a split tangential G mode located at ∼1580 cm^−^^1^. This band represents the sp^2^ in-plane bond-stretching of C atoms in graphite-like structures. The D mode at ∼1350 cm^−^^1^ becomes evident if sp^3^ C atoms are introduced in the sp^2^ carbon lattice [31]. The radial breathing mode (RBM) between 130 and 300 cm^−^^1^ corresponds to the coherent vibration of the C atoms in the radial direction of the nanotube and it is shown in the 100–350 cm^−1^ window in Figure 5, panel A. For the functionalized samples (solid lines) we found a general decrease in the RBM intensities of the lower frequency side. We interpreted this behavior as an indication of changes in the electronic structure of the tubes.

From the intensity ratio of the Raman D and G modes (AD/AG ratio) [32] it is possible to estimate the defect concentration in SWCNTs. The AD/AG ratios were corrected by the AD_0_/AG_0_ values of not modified MX@SWCNT material. The D and G bands of compounds **2** and **4** are shown together (Figure 5, panel B).

The D mode increased with the increased defects concentration and it is much higher after functionalization with amine in comparison to starting material MX@SWCNTs. The (AD/AG)/(AD_0_/AG_0_) ratios are compared to the functionalization degrees calculated from the weight loss of the TGA and are shown in Table 1.

**Figure 6:**
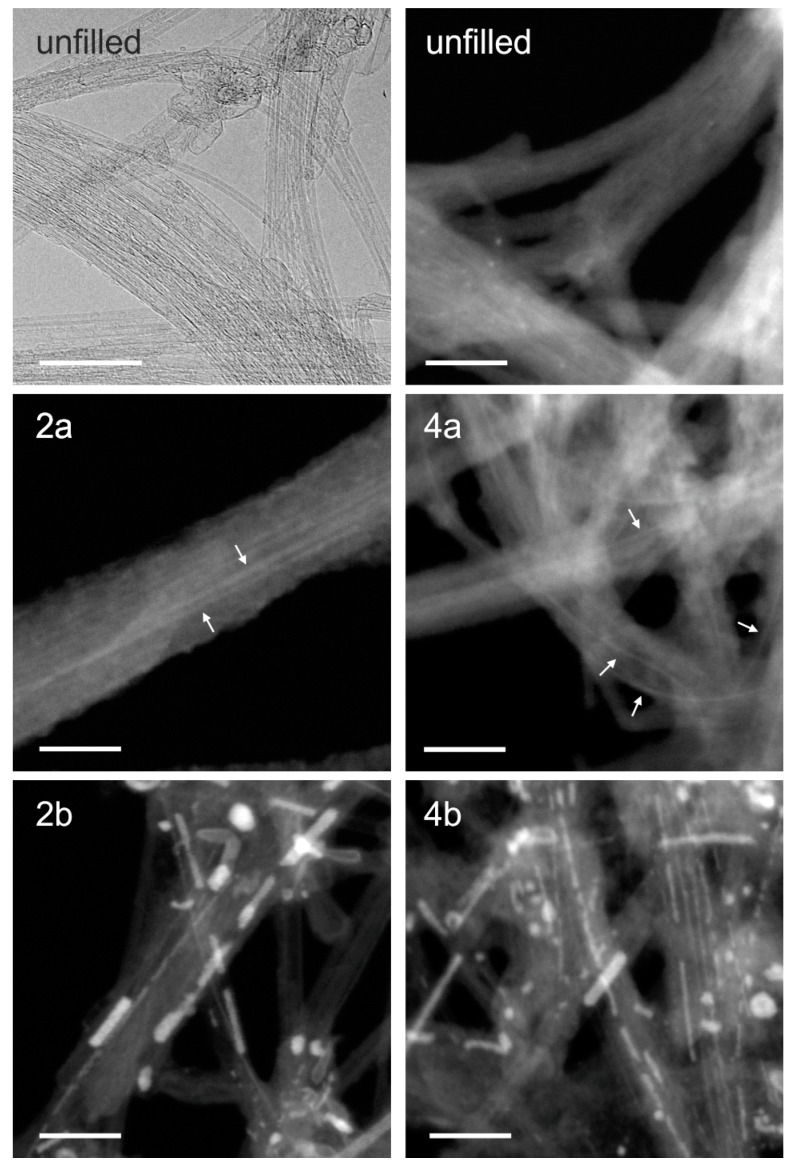
HRTEM and HAADF-STEM images of unfilled SWCNTs (first row); HAADF-STEM images of **2a**, **4a**, **2b** and **4b**. Scale bar: 20 nm.

HAADF-STEM images taken on the filled SWCNTs **2a** and **2b** show the presence of the encapsulated metal halides inside the tubes, as visible from Figure 6. In this imaging mode, the intensity is approximately proportional to the square of the atomic number; therefore the bright lines that follow the shape of the CNTs correspond to the filling material. This contrast is not observed in the HAADF-STEM image of the unfilled tubes (Figure 6, first row). Remarkably, when comparing the filled tubes **2a** and **2b** with their functionalized counterparts **4a** and **4b**, the same degree of filling is observed. Therefore it can be concluded that the filling is preserved upon functionalization.

**Figure 7:**
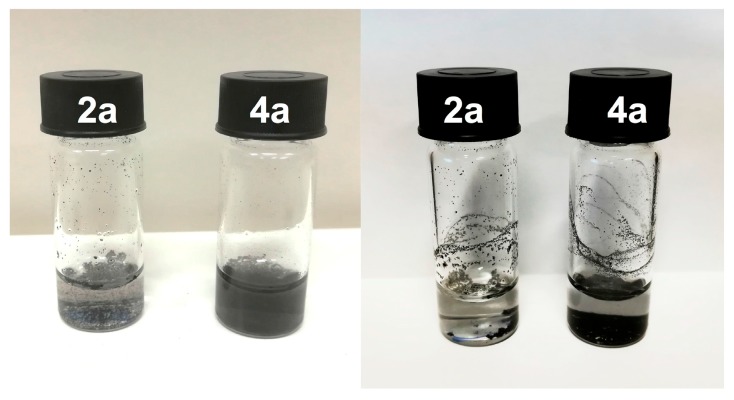
Suspensions of **2a** and **4a** in H_2_O (1 mg·mL^−1^) just after sonication (left panel) and 10 min later (right panel).

Even though the purpose of this work was to demonstrate the applicability of this procedure to gently oxidize the tubes while preserving their content, the use of such strategy allowed also to obtain materials with better water-dispersibility. In a qualitative experiment 1 mg of **2a** and of **4a** were dispersed in 1 mL of distilled water and sonicated for 2 min. As appreciable from Figure 7 a better suspension was obtained for **4a**, but in both cases the compounds settled to the bottom in for 10 min.

## 4. Conclusions

In summary, we report on the decoration of closed-ended filled carbon nanotubes (carbon nanocapsules) with epoxide groups under mild conditions by TFDO. This reaction does not necessitate of metal catalyst and maintained the CNT structure, in particular the closed ends of the nanotubes during treatment. Remarkably, in this procedure there is no need of an oxidizing acid treatment that would damage the structure of the nanotubes, especially on the tips, during the oxidation process. In this way the tips stay sealed while for instance nitric acid treatment of filled SWCNTs results in the complete leakage of the encapsulated metal halides because the ends are re-opened.

The subsequent transformation of the epoxides introduced on the *ox*-MX@SWCNT is obtained with the epoxide ring-opening by means of a primary diamine. Such an approach highlights the versatility of the *ox*-MX@SWCNT as valuable platforms for their successive functionalization/derivatization with selected reactive nucleophiles in addition to providing multimodality to the final derivatives.

## Figures and Tables

**Table 1:** Screening of different SWCNTs filled with biomedical relevant payloads and modified by oxidation and amination.

Products	Weight Loss ^1^ (%)	f ^2^ (µmol/g)	(AD/AG)/(AD_0_/AG_0_) ^3^
*f*-NaI@SWCNTs (**4a**)	9.7	730	1.8
*f*-LuCl_3_@SWCNTs (**4b**)	11.9	900	2.1

^1^ Weight loss obtained at 500 °C. ^2^ Degree of functionalization f (µmol/g) calculated from the measured weight loss. ^3^ (AD/AG)/(AD_0_/AG_0_) ratios obtained from Raman spectroscopy at 633 nm.
